# Analysis of the causes of neonatal death and genetic variations in congenital anomalies: a multi-center study

**DOI:** 10.3389/fped.2024.1419495

**Published:** 2024-08-14

**Authors:** Xue Yang, Ruimiao Bai, Juan Zhang, Yunfan Yang, JuanJuan Zhang, Baozhu Wang, Zhankui Li, Xiping Yu

**Affiliations:** ^1^ Department of Health, Northwest Women’s and Children’s Hospital, Xi’an, Shaanxi, China; ^2^ Department of Neonatology, Northwest Women’s and Children’s Hospital, Xi’an, Shaanxi, China

**Keywords:** mortality rate, cause of death, congenital anomalies, inherited metabolic disorders, genetic testing

## Abstract

**Background:**

Neonatal deaths often result from preventable conditions that can be addressed with appropriate interventions. This study aims to analyze the distribution of the causes of neonatal death and explore genetic variations that lead to congenital anomalies in Northwest China.

**Methods:**

This multi-center observational study was conducted across six medical centers in Shaanxi province, Northwest China. Clinical data were retrospectively collected from neonates admitted between 2016 and 2020. Kaplan-Meier analysis was utilized to estimate survival rates, while high-throughput sequencing platforms were employed to detect mutations causing congenital anomalies.

**Results:**

Among 73,967 neonates requiring hospital care, 424 neonatal deaths were recorded, leading to a neonatal mortality rate of 0.57%. The primary causes of death included neonatal respiratory distress syndrome (23.8%), birth asphyxia (19.8%), neonatal septicemia (19.3%), and congenital anomalies (13.6%). The leading causes of neonatal deaths due to congenital anomalies were congenital heart defects (38.6%), bronchopulmonary dysplasia (14.0%), and inherited metabolic disorders (10.5%). Genetic analysis identified 83 pathogenic or likely pathogenic variants in 23 genes among the neonates with congenital anomalies, including four novel mutations (c.4198+1G>T, c.1075delG, c.610-1G>A, c.7769C>T) in the *ABCC8*, *CDKL5*, *PLA2G6*, and *NIPBL* genes.

**Conclusion:**

Congenital anomalies represent a significant and preventable cause of neonatal deaths in Northwest China. Early detection of congenital anomalies through genetic testing and comprehensive prenatal care are crucial for reducing neonatal mortality rates and improving pregnancy outcomes.

## 1 Introduction

Globally, there were 2.44 million neonatal deaths in 2019, constituting nearly half (47%) of all deaths among children under 5 years of age (1). Most of these deaths occurred in developing countries (2). Despite a 55.6% decrease in neonatal deaths in China over the past decade, approximately 64,000 neonates died in 2019, translating to a mean value of seven neonatal deaths per hour (1).

According to recent research, neonatal deaths often result from complications during labor, preterm birth, and congenital anomalies (3, 4). Although advances in prenatal and neonatal healthcare have markedly improved outcomes for adverse perinatal conditions, the survival rates for congenital anomalies have not shown equivalent progress (5). Congenital anomalies frequently lead to severe complications, such as metabolic decompensation, rapid clinical deterioration, intellectual disability, developmental delays, and mortality (6). However, many of these deaths are preventable through effective interventions, such as neonatal screening, genetic testing, and appropriate treatment (3, 6, 7).

The “Healthy China 2030” initiative emphasizes the enhancement of congenital anomaly prevention and control systems to improve newborn health. Addressing congenital anomalies is crucial for meeting the targets of Sustainable Development Goals (SDGs) 3.2.1, which aim to eliminate preventable neonatal and under-5 child deaths by 2030 (8). Therefore, we conducted a multicenter observational study to analyze the distribution of the causes of neonatal death and explore the genetic variations that lead to congenital anomalies in Northwest China. Our objective was to propose targeted intervention strategies aimed at reducing neonatal fatalities.

## 2 Materials and methods

### 2.1 Study design and population

**Figure 1:**
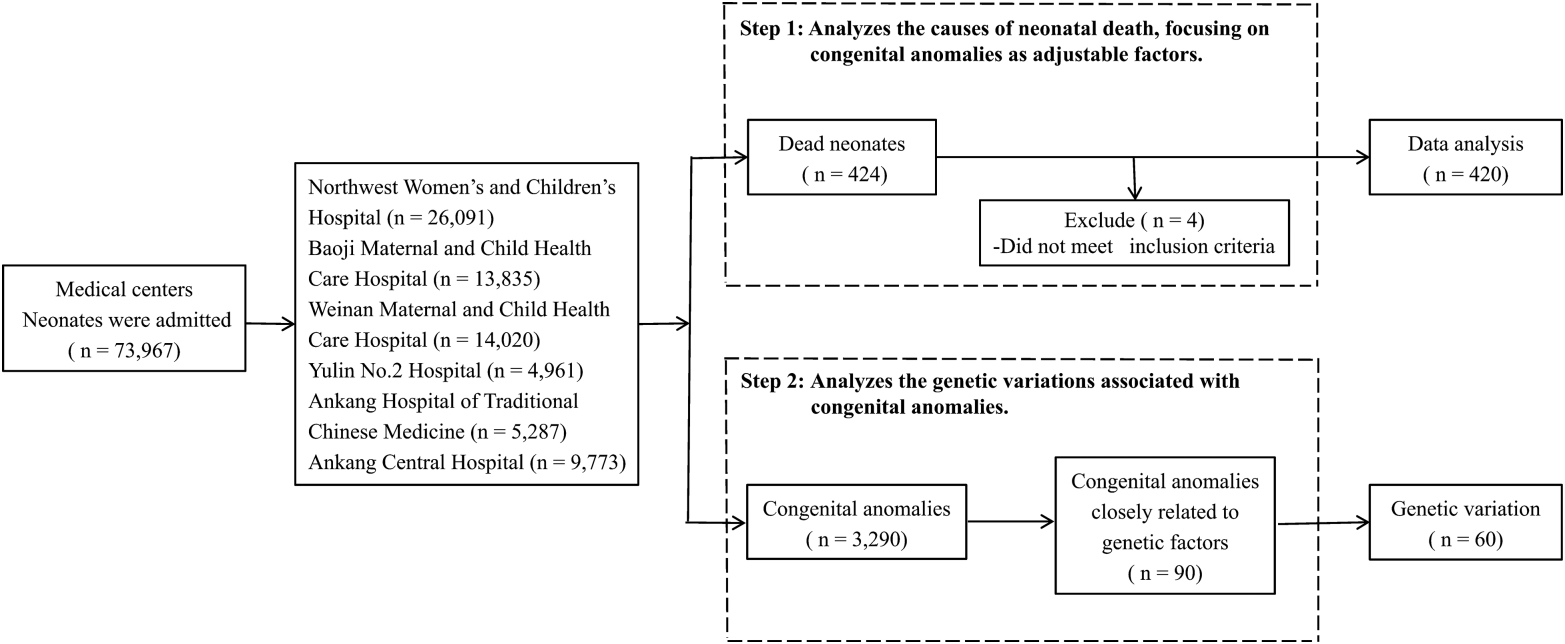
Participant flow chart.

This study was conducted in Shaanxi province, Northwest China, employing stratified and cluster sampling methods to ensure a geographically balanced representation. From the 10 districts within Shaanxi, a total of five cities and six medical centers were selected: Northwest Women's and Children's Hospital, Baoji Maternal and Child Health Care Hospital, Weinan Maternal and Child Health Care Hospital, Yulin No. 2 Hospital, Ankang Hospital of Traditional Chinese Medicine, and Ankang Central Hospital (Figure 1). These centers include provincial and municipal rescue transit centers for critically ill newborns.

Technical investigators, trained to deliver high levels of consistency, were stationed at each center for data collection. Clinical data were extracted retrospectively from medical records, encompassing variables such as birth weight (BW), gestational age (GA), sex, Apgar score, biochemical indicators, clinical causes of death, and other relevant information. Maternal data were retrieved through a retrospective review of antenatal care records, covering demographic characteristics, antenatal and perinatal history, and delivery conditions.

Our dataset includes all liveborn neonates hospitalized in the neonatology departments of these centers between 2016 and 2020. During this period, 220,488 neonates were born in these centers, constituting 12% of all live births in the province. Of these, 73,967 neonates required hospital care, and 424 neonatal deaths were recorded. Stillbirths and neonates born before the gestational age of 24 weeks were excluded from our analysis. Among neonates with congenital anomalies, genetic testing was conducted in 90 cases that were selected based on a strong suspicion of underlying genetic variations or clinical complexities hindering a definitive diagnosis via conventional methods. Final diagnoses were achieved through a comprehensive genetic analysis.

### 2.2 Various definitions

Neonatal diseases were diagnosed according to the standards and definitions described in Practical Neonatology (9) and categorized according to the International Classification of Diseases, Tenth Revision (10), as assessed by pediatricians. For analysis, causes of death other than neonatal respiratory distress syndrome (NRDS), birth asphyxia, neonatal septicemia, congenital anomalies, pneumorrhagia, and meconium aspiration syndrome (MAS) were labeled as “others.”

GA was estimated based on a maternal history of weeks since the first day of the last menstrual period. Preterm birth was defined as neonates born alive before 37 weeks of pregnancy, with extremely preterm birth defined as birth before 28 completed weeks of gestation. Low birth weight (LBW) was denoted as a BW <2,500 g, and very LBW as a BW <1,500 g. Maternal diseases during pregnancy included hypertensive disorders complicating pregnancy, gestational diabetes mellitus, gestational hypothyroidism, and other diagnosed conditions.

### 2.3 Genetic analysis

Genomic DNA was extracted from peripheral blood samples of neonates and their parents. Mutation detection utilized a high-throughput sequencing platform, chromosome karyotype analysis, and chromosomal microarray. Sanger sequencing was employed to verify the mutated sequences (conducted by Giant Medical Diagnostics Institute, Cipher Gene Institute, and Kingmed Diagnostics Institute).

### 2.4 Statistical analysis

Data completeness was ensured through manual checks and double entry into a data management system, with logical checks for range and accuracy. Continuous variables were summarized using arithmetic mean and standard deviation, while categorical variables were presented as frequencies and proportions. Bar graphs depicted neonatal deaths by BW and GA, and pie charts illustrated the distribution of congenital anomalies. Neonatal death rates were calculated using hospitalized neonates as the denominator. Chi-square tests were utilized to analyze categorical data. Kaplan–Meier methods were applied to estimate the survival rate of neonates over time. All statistical analyses were performed using SPSS software version 23.0 (IBM Corp., Chicago, IL). Two-tailed tests of statistical significance were applied universally, with the significance level set at 5% (*P* < 0.05).

### 2.5 Ethics approval

This study was approved by the Ethical Committee of Northwest Women's and Children's Hospital (Ethical Approval Number: 2022002). Written informed consent to participate in this study was provided by the legal guardians of the patients. Patient anonymity and confidentiality were ensured.

## 3 Results

### 3.1 Demographic characteristics

**Figure 2:**
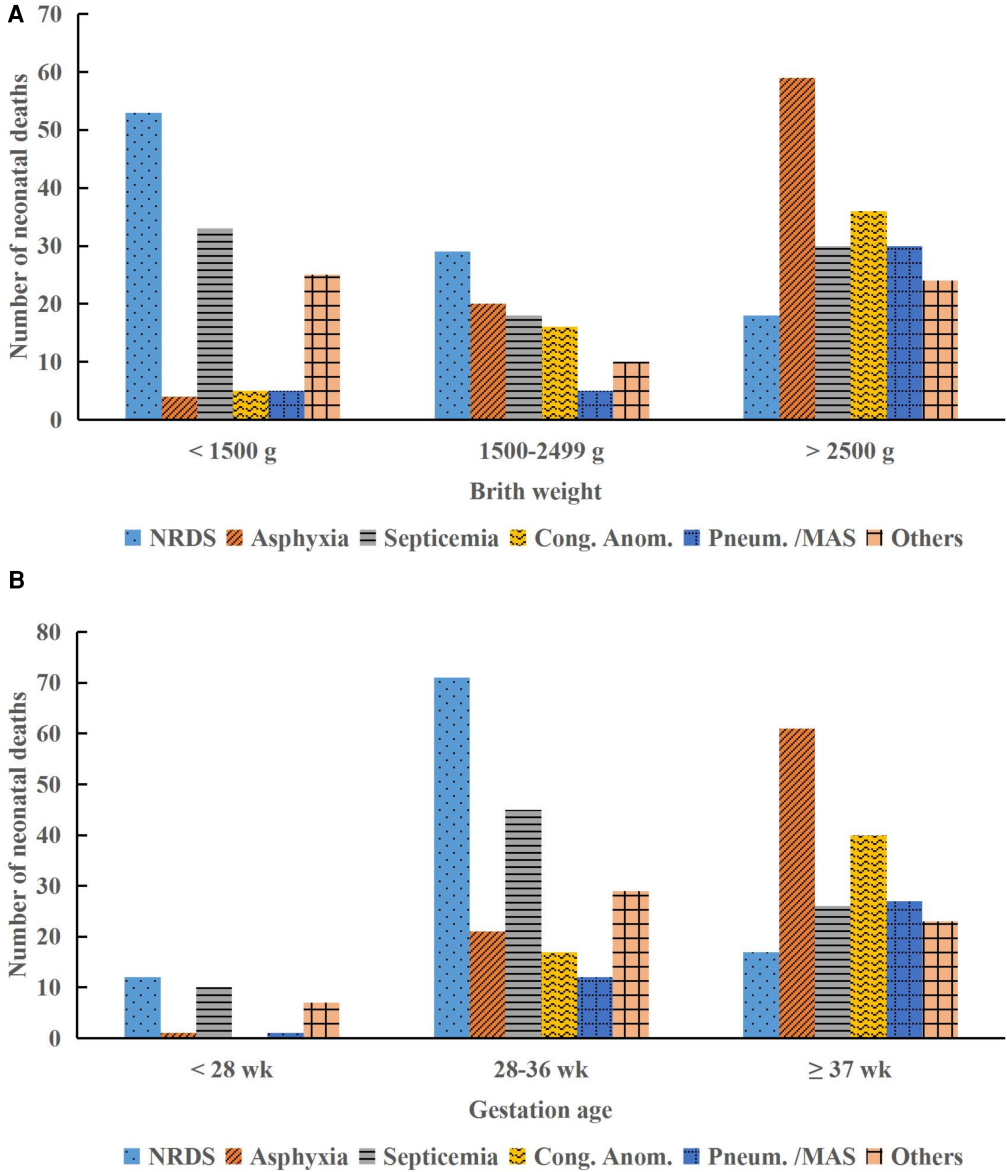
Distribution of neonatal deaths by birth weight and gestational age categories. **(A)** Causes of death by birth weight. **(B)** Causes of death by gestational age. NRDS, neonatal respiratory distress syndrome; Cong. Anom., congenital anomalies; Pneum., pneumorrhagia; MAS, meconium aspiration syndrome; g, gram; wk, week.

Among the 73,967 neonates requiring hospital care, 420 neonates died, resulting in a mortality rate of 0.57%. The majority of deaths occurred in the first week of life (76.9%), with a higher incidence observed in infant boys (55.7%) compared to infant girls (44.3%). LBW was observed in 53.1% of neonates, and preterm births accounted for 53.8% of all neonatal deaths. The primary causes of neonatal deaths included NRDS (100/420, 23.8%), birth asphyxia (83/420, 19.8%), neonatal septicemia (81/420, 19.3%), congenital anomalies (57/420, 13.6%), and pneumorrhagia/MAS (40/420, 9.5%), accounting for 86.0% (361/420) of all neonatal deaths (Table 1). Figure 2 illustrates that deaths due to birth asphyxia increased significantly with a rise in BW and GA, peaking in neonates with a BW >2,500 g and a GA ≥37 weeks (Chi-squared test, *P* < 0.001). Similarly, most of the neonatal deaths attributed to congenital anomalies occurred in normal-term and normal BW neonates, accounting for 70.2% (40/57) and 63.2% (36/57) of cases, respectively (Chi-squared test, *P* < 0.001).

**Table 1:** Summary of maternal and neonatal characteristics among the deceased neonates, 2016–2020 (*n* = 420).

Variable	*N* (%)/mean ± SD
Maternal antenatal and perinatal characteristics	
Age at delivery, mean ± SD (years)	29.2 ± 4.9
Mode of delivery	
Vaginal delivery	185 (44.0)
Cesarean section	235 (56.0)
Maternal disease	
No	275 (66.1)
Yes	141 (33.9)
Neonatal birth outcomes and death status	
Gestation at birth, mean ± SD (weeks)	35.0 ± 4.8
Birth weight, mean ± SD (g)	2,292.7 ± 991.8
Apgar score at 1 min after birth, mean ± SD	5.7 ± 3.0
Apgar score at 5 min after birth, mean ± SD	6.9 ± 2.9
Sex, *n* (%)	
Male	234 (55.7)
Female	186 (44.3)
Death age	
<24 h	160 (38.1)
1–6 days	163 (38.8)
≥7 days	97 (23.1)
Death place	
Hospital	293 (69.8)
Way to hospital	91 (21.7)
Home	36 (8.6)
Cause of death	
NRDS	100 (23.8)
Birth asphyxia	83 (19.8)
Neonatal septicemia	81 (19.3)
Congenital anomalies	57 (13.6)
Pneumorrhagia/MAS	40 (9.5)
Others	59 (14.0)

MAS, meconium aspiration syndrome; NRDS, neonatal respiratory distress syndrome; SD, standard deviation.

### 3.2 Survival status of neonates over time

**Figure 3:**
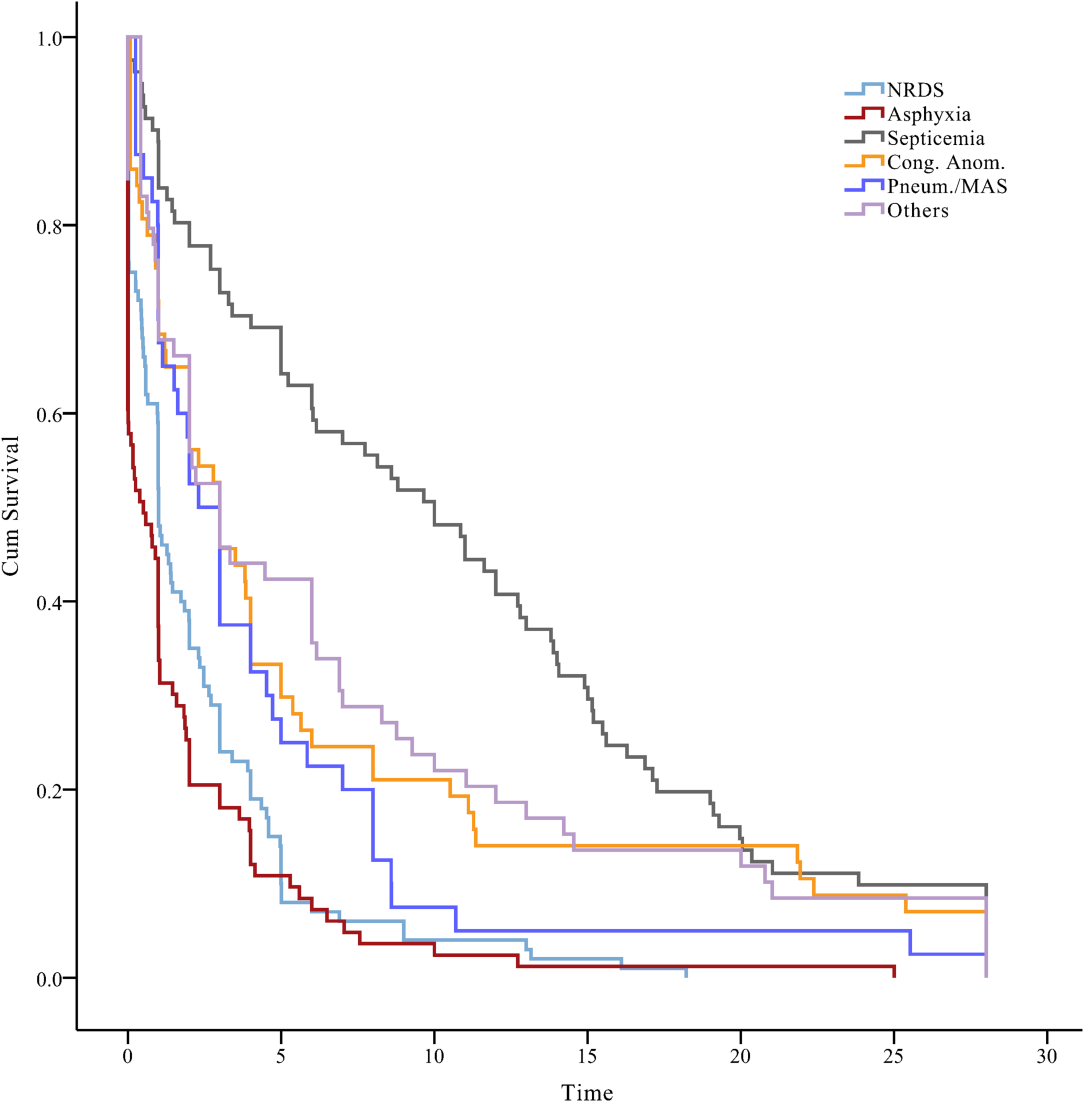
A plot of the status of neonates over time, beneath the Kaplan–Meier plot. NRDS, neonatal respiratory distress syndrome; Cong. Anom., congenital anomalies; Pneum., pneumorrhagia; MAS, meconium aspiration syndrome.

The temporal trends in our survival status data indicated that neonates diagnosed with neonatal septicemia had the longest median event-free survival time of 10.7 days [95% confidence interval (CI), 8.8–12.6]. Congenital anomalies exhibited a median survival time of 6.2 days (95% CI, 4.0–8.4), while birth asphyxia had the shortest median survival time of 1.8 days (95% CI, 1.0–2.5) (log-rank test, *P* < 0.001) (Figure 3). Early identification and intervention in congenital anomalies, compared with conditions such as birth asphyxia, may lead to improved survival outcomes for neonates.

### 3.3 Congenital anomalies

**Figure 4:**
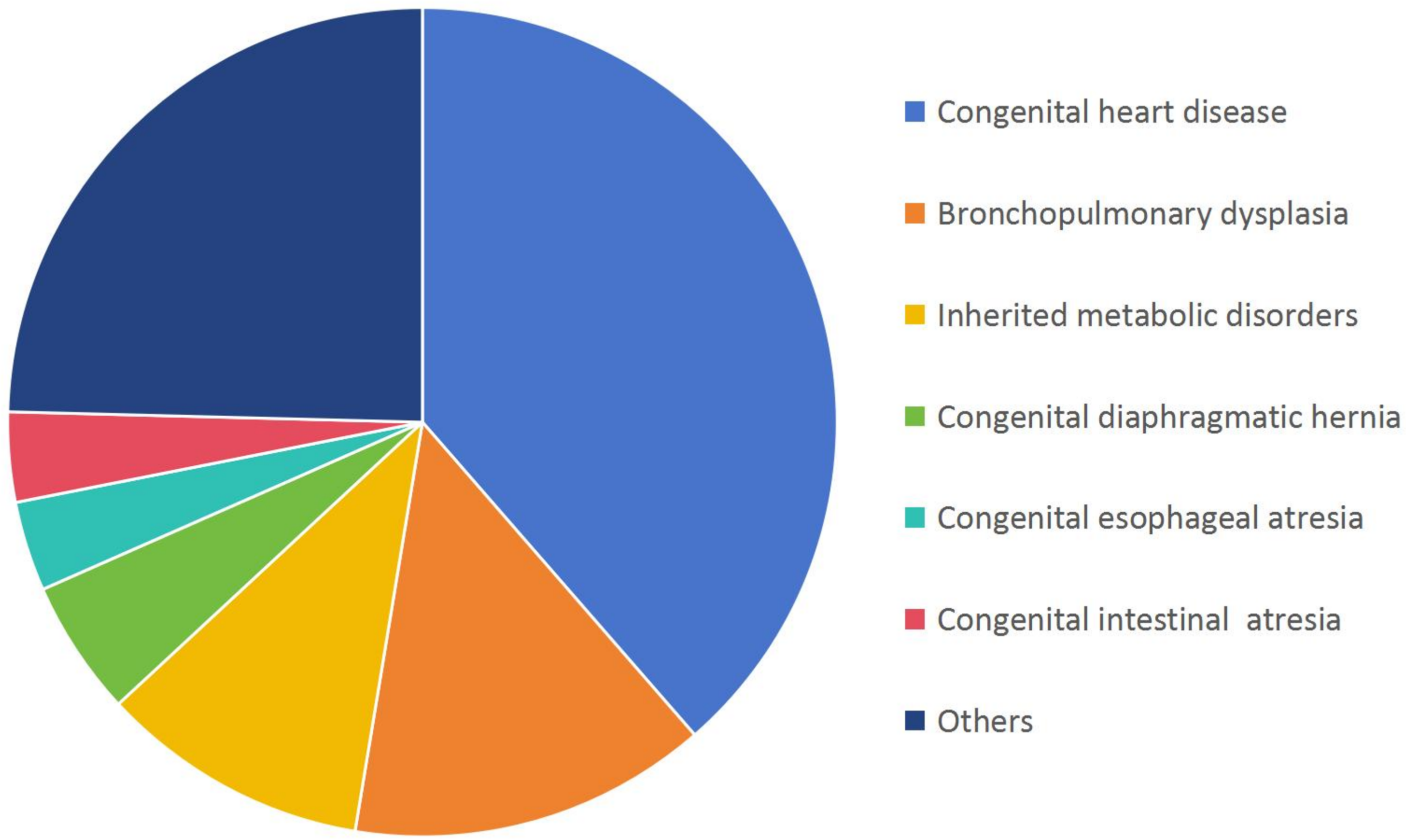
Distribution of congenital anomalies in deceased neonates (*n* = 57).

Congenital heart defects (CHDs, 22/57, 38.6%), bronchopulmonary dysplasia (8/57, 14.0%), and inherited metabolic disorders (IMDs, 6/57, 10.5%) were the predominant causes of neonatal deaths attributed to congenital anomalies. Among neonatal deaths attributed to IMDs, methylmalonic acidemia had the highest proportion (2/6), followed by glutaric acidemia (1/6), hyperammonemia (1/6), mitochondrial diseases (1/6) and congenital amino acid metabolic disease (1/6), and all of them are closely related to genetic factors (Figure 4). Genetic mutations play a critical role in the pathogenesis of many congenital anomalies, emphasizing the importance of genetic testing in neonatal care.

### 3.4 Genetic testing results

Genetic variants were identified in 60 neonates with congenital anomalies. The majority were infant boys (58.3%), with a mean GA of 38.9 ± 1.7 weeks and a mean BW of 3,188.4 ± 552.8 g. Amino acid metabolic disorders accounted for the most common genetic variants, representing 41.6% (25/60) of cases (Table 2).

**Table 2:** Pathogenic or likely pathogenic variants among 60 neonates with congenital anomalies, 2016–2020 (*n* = 60).

Disease	Gene	Chromosome location	Variants	Number of carriers, *n* (%)
Phenylketonuria				
	*PAH*	chr12	c.194T>C(p.Ile65Thr), c.208–210delTCT(p.Ser70del), c.284_286delTCA(-), c.331C>T(p.Arg111*), c.331C>T(p.Arg111*), c.440C>T(p.Pro147Leu), c.611A>G(p.Y204C), c.611A>G(p.Y204C), c.688G>A(p.Val230Ile), c.728G>A(p.R243Q), c.907delT(p.S303Pfs*38), c.1024G>A(p.Ala342Thr), c.1068C>A(p.Y356X), c.1197A>T(p.Val399Val), c.1197A>T(p.Val399Val), c.1222C>T(p.R408W), c.1223G>A(p.Arg408Gln), c.1229T>C(p.Phe410Ser), c.1252A>C(p.T418P)	13 (21.7)
Hyperphenylalaninemia				
	*PAH*	chr12	c.442-1G>A(splicing), c.689T>C(p.V230A), c.473G>A(p.Arg158Gln), c.838G>A(p.Glu280Lys)	2 (3.3)
Methylmalonic acidemia				
	*MUT*	chr6	c.323G>A(p.R108H), c.729_730insTT(p.Asp244fs), c.1233_1235delCAT (p.1412del), c.1531C>T(p.Arg511*), c.1630G>T (p.Gly544*)	3 (5.0)
	*MMACHC*	chr1	c.271dupA(p.Arg91Lysfs*13), c.609G>A(p.Trp203*), c.566delinsGT(p.I190Yfs*13), c.609G>A(p.W203X)	2 (3.3)
Homocystinemia				
	*MMACHC*	chr1	c.331C>T(p.Arg111*), C.445_446delTG(p.Cys149fs), c.567dupT(p.1190Yfs*13), c.609G>A(p.W203X), c.609G>A(p.Trp203*), c.394C>T(p.Arg132*), c.482G>A(p.R161Q), c.609G>A(p.Trp203Ter)	5 (8.3)
Progressive muscular dystrophy				
	*DMD*	chrX	exon(3-13)del, exon45del, exon51del, exon(10-11)del, exon(44-50)del	5 (8.3)
Spinal muscular atrophy				
	*SMN1*	chr5	exon7del, exon(7-8)del, exon(7-8)del	3 (5.0)
Prader–Willi syndrome				
	*SNRPN*	chr15	15q11.2-q13del, 15q11.2-q13del, 15q11-q13del	3 (5.0)
Epileptic encephalopathy				
	*CDKL5*	chrX	c.1075delG(p.Gly359Valfs*9)^a^, c.1375C>T(p.Gln459*), c.1375C>T(p.Gln459*)	3 (5.0)
Congenital hyperinsulinemia				
	*ABCC8*	chr11	c.824G>A(p.R275Q), c.1887delC(p.T630Hfs*17), c.2921-9GC>A(-), c.3022_3028delGGCATC(p.Gly1008fs), c.4198+1G>T(splicing)^a^	2 (3.3)
Congenital adrenal hyperplasia				
	*CYP21A2*	chr6	c.293-13C>(-), c.293-13C>(-)	2 (3.3)
Infantile neuroaxonal dystrophy				
	*PLA2G6*	chr22	c.1978C>T(p.P660S), c.610-1G>A(-)^a^	1 (1.7)
Cornelia de Lange syndrome Ⅰ				
	*NIPBL*	chr5	c.7769C>T(p.P2590l)^a^	1 (1.7)
Tuberous sclerosis complex				
	*TSC2*	chr16	c.5127dupC(p.Thr1674Thrfs*31)	1 (1.7)
Congenital ectodermal dysplasia syndrome				
	*EDA*	chrX	c.463C>T(p.Arg155Cys)	1 (1.7)
P450 oxidoreductase deficiency				
	*POR*	chr7	c.1323fs*8(p.Pro442Profs*8), c.1370G>A(p.Arg457His)	1 (1.7)
Gangliosidosis				
	*GLB1*	chr3	c.1343A>T(p.Asp44 8Val), c.785G>T(p.Gly26 2Val)	1 (1.7)
Multiple carboxylase deficiency				
	*HLCS*	chr21	c.789delG(p.Gly261fs), c.1522C>T(p.Arg508Trp)	1 (1.7)
Down syndrome				
	—	—	47, XY, +21, 47, XY, +21	2 (3.3)
Others				
	—	—	*UGT1A1*, *EDA*, *SCN2A*, *RANBP2*, *SCN8A*, *DSG2*, 47, XX, 47, XX	8 (13.3)

^a^ Novel mutations.

A total of 83 pathogenic or likely pathogenic variants were identified in 23 genes, including *PAH*, *MUT*, *MMACHC*, *CYP21A2*, *ABCC8*, *SNRPN*, *CDKL5*, *DMD*, *SMN1*, *PLA2G6*, *NIPBL*, *TSC2*, *EDA*, *POR*, *GLB1*, *HLCS*, *UGT1A1*, *SCN2A*, *RANBP2*, *SCN8A*, *DSG2*, 15q11del, and 5q13del. These genes exhibited an autosomal dominant or recessive inheritance pattern in 17 cases (73.9%), while 6 cases (26.1%) followed an X-linked dominant or recessive inheritance pattern. In addition, four novel mutations (c.4198+1G>T, c.1075delG, c.610-1G>A, c.7769C>T) were identified in the *ABCC8*, *CDKL5*, *PLA2G6*, and *NIPBL* genes, respectively. A case of congenital hyperinsulinemia (CHI) involved two mutations in the *ABCC8* gene, namely, c.2921-9 G>A (chr11:17428685) and c.3022_3028delGGCATC (chr11:17428568), demonstrating successful outcomes with tailored treatment guided by genetic testing.

## 4 Discussion

We observed an overall neonatal mortality rate of 0.57% which is relatively low for Shaanxi province, China, and comparable to the rates reported in developed countries (1). Geographic location, ethnicities, and cultural practices influence the causes of neonatal deaths (11), though certain fundamental causes persist universally. The primary causes of neonatal mortality identified by our present findings included NRDS, birth asphyxia, neonatal septicemia, congenital anomalies, and pneumorrhagia/MAS, and our data align with the globally recognized leading causes of neonatal death (1).

Over three-quarters (76.9%) of neonatal deaths occurred within the first week of life, with nearly half occurring within the first 24 h. A significant majority (58.8%) of deceased neonates experienced adverse birth outcomes such as preterm birth or LBW, which was slightly higher than that reported in similar studies (12–14). Most hospitals in our study served as rescue transit centers, admitting numerous high-risk pregnancies, which likely contributed to the elevated preterm birth rates observed.

NRDS was the predominant cause of neonatal death, predominantly affecting preterm (83%) and LBW (82%) neonates, with most fatalities occurring within the first 7 days of life (94%). This finding is consistent with studies in developing countries (15, 16), reporting an NRDS contribution to death rates of 45%–63% among preterm neonates. Birth asphyxia accounted for nearly one-fifth of all neonatal deaths with a mean survival time of only 1.8 days. In comparison, an Ethiopian study reported that birth asphyxia accounted for 14% of neonatal deaths, whereas a Kenyan study reported 32% of neonatal deaths to be from this cause (17). These discrepancies may arise from variations in the healthcare infrastructure and quality across different geographic regions.

Congenital anomalies accounted for approximately 13.6% of neonatal deaths in our study, and this is a lower proportion than the 30%–40% reported in European and American studies (18, 19), but is consistent with findings from East China (12). Although multifactorial in nature, previous studies have highlighted the close association between IMDs and genetic factors (20). A timely intervention can improve outcomes in >80% of newborns with non-lethal anomalies (21), highlighting the importance of enhanced maternal screening and early post-birth interventions.

Genetic testing plays a crucial role in identifying cases of CHI caused by mutations in the *ABCC8* gene. Personalized treatment strategies guided by genetic testing can effectively manage this condition, emphasizing their significance in neonatal care. *ABCC8* mutations impair trafficking of the sulfonylurea receptor 1 (SUR1) subunit to the plasma membrane or hinder channel activity, leading to persistent membrane depolarization, potassium (K^+^) channel closure, prolonged calcium (Ca^2+^) channel opening, and excessive insulin secretion. Neonatal hyperinsulinemia poses the risk of irreversible hypoglycemic brain injury or death (22, 23) and is managed effectively with diazoxide, an adenosine triphosphate-sensitive K^+^ channel opener, in combination with SUR1 regulation (24).

However, genetic testing utilization remains limited in Shaanxi province owing to inadequate laboratories for single-gene tests and gene panels in medical centers, highlighting a critical gap in healthcare. Enhancing access to neonatal genomic medicine services is essential for improving diagnostic capabilities and treatment outcomes (25). Therefore, we should consider placing a stronger emphasis on neonatal screening and genetic testing as key strategies for achieving a marked reduction in neonatal mortality rates, and this could significantly help reduce the burden of these disorders in populations, especially those in developing countries.

Our study has several limitations. First, the study was conducted exclusively in Shaanxi province, located in northwestern China, and this geographic region may not fully represent the national situation in China. Shaanxi province has a population of approximately 36 million, and many residents live in economic poverty. The six participating medical centers were evenly distributed across this region, suggesting a sufficiently representative sample that reflected the situation of neonates in this disadvantaged area of China. Second, although our study offers valuable insights into clinical practices, the data were primarily derived from high-level rescue transit medical centers that cater to high-risk pregnant women and critically ill neonates transferred from local hospitals. Therefore, caution must be exercised when interpreting these data. Overall, population-based surveillance is required to comprehensively monitor the incidence of congenital anomalies and inform public health interventions.

In conclusion, congenital anomalies are a significant cause of neonatal death in Northwest China, warranting primary public health attention. Our findings underscore a crucial opportunity for intervention, suggesting that many deaths attributed to congenital anomalies may be preventable or treatable by using appropriate measures. To substantially reduce neonatal mortality, comprehensive initiatives should prioritize antenatal care, neonatal screening programs, and genetic testing as essential components of long-term prevention strategies.

## Data Availability

The datasets presented in this study can be found in online repositories. The names of the repository/repositories and accession number(s) can be found in the article/Supplementary Material.
